# Aphonia of Four Years’ Standing, Cured by Electro-Magnetism

**Published:** 1854-01

**Authors:** F. K. Bayley

**Affiliations:** Almont


From the Peninsular Journal of Medicine.

*Aphonia of Four Years' Standing, cured by Electro-Magnetism.* By F. K. BAYLEY, M.D., Almont.

Mrs. S——, aged 78, in the spring of 1849, had a severe attack of Bronchitis, which was relieved by appropriate treatment.

On regaining her general strength, however, her voice was at times very hoarse, and at the close of the day it was difficult to speak loud at all. In the course of six months from the first attack, there was a complete Aphonia, which continued until last April.

At that time she was induced to make trial of Electro-Magnetism. In a few days after this means was tried, her voice became more distinct, but very rough at first. In the course of a week or two speech was natural, and has continued until the present time.

The favorable result in this case may lead to the use of Electro-Magnetism in other affections produced by want of proper innervation. I will add, the apparatus used was one manufactured by Charles Crosman, Detroit.

October, 1853.

